# Pulmonary Functions and Capacities and Their Associations with Sociodemographic, Physical and Behavioural Risk Factors in Firefighters

**DOI:** 10.3390/ijerph23030370

**Published:** 2026-03-14

**Authors:** Jaron Ras, Jordan Sasha Kaylor, Lloyd Leach

**Affiliations:** Department of Sport, Recreation and Exercise Science, Faculty of Community and Health Sciences, University of the Western Cape (UWC), Private Bag X17, Bellville, Cape Town 7535, South Africa; 4325341@myuwc.ac.za (J.S.K.); lleach@uwc.ac.za (L.L.)

**Keywords:** spirometry, respiratory health, obesity, smoking, physical activity’ occupational exposure

## Abstract

**Highlights:**

**Public health relevance—How does this work relate to a public health issue?**

- This study addresses occupational respiratory health among firefighters, a critical public health issue given their routine exposure to smoke, particulate matter, and toxic chemicals that place them at elevated risk of acute and chronic pulmonary disease.
- By documenting the prevalence of respiratory symptoms and changes in pulmonary function in a South African firefighting cohort, this study contributes to understanding the health burden associated with emergency service occupations.

**Public health significance—Why is this work of significance to public health?**

- Firefighters represent an essential workforce, and declines in pulmonary function linked to occupational exposure, smoking, physical inactivity, ageing, and adverse body composition have implications for workforce sustainability, healthcare utilisation, and long-term morbidity.
- The findings provide population-specific evidence from a low- and middle-income country context, addressing a notable gap in the global occupational health literature and informing locally relevant prevention strategies.

**Public health implications—What are the key implications or messages for practitioners, policy makers and/or researchers in public health?**

- For practitioners and policymakers, the results highlight the need for routine pulmonary function surveillance, smoking cessation support, targeted fitness programmes, and body composition management as part of comprehensive occupational health policies for firefighters.
- For researchers, this study highlights the importance of longitudinal investigations into cumulative occupational exposure, ageing, and modifiable lifestyle factors to inform evidence-based interventions that protect respiratory health in high-risk occupations.

**Abstract:**

Introduction: Firefighters are exposed to toxic smoke and hazardous environmental conditions that place them at risk for pulmonary disorders. This study aimed to determine the prevalence of pulmonary symptoms and disorders among full-time firefighters in the City of Cape Town Fire and Rescue Service and to explore associations with sociodemographic, physical and behavioural risk factors. Methods: A cross-sectional, quantitative study was conducted among 278 full-time firefighters using convenience sampling. Data were collected using a researcher-generated questionnaire and spirometry measurements. Physical characteristics were assessed using bioelectrical impedance analysis. Descriptive and inferential statistics were applied (Kruskal–Wallis H, Chi-squared test, Pearson’s correlation), with *p* < 0.05 denoting statistical significance. Results: More than half (53.60%) of firefighters presented with at least one pulmonary symptom. Coughing (54.36%) and nasal congestion (40.94%) were the most common symptoms. Pulmonary symptoms were more frequent among firefighters aged 30–49 years. Significant differences were found in pulmonary function between age categories (*p* < 0.01) and obesity in firefighters (*p* < 0.01). Negative correlations were found between FVC and BMI (r =−0.35), BG% (r = −0.47) and years of experience (r =−0.21). Conclusions: Findings highlight the occupational burden of pulmonary health risks and the need for regular screening and preventive strategies within firefighting populations.

## 1. Introduction

Occupational exposure in firefighting is complicated and covers a range of risks from both fire and non-fire situations [1]. These exposures include inhalation of toxic chemicals and fumes, from wild, structural and chemical fires, while also attending to duties such as traffic collisions, emergency resuscitation and emergency rescues [2,3,4,5,6]. Firefighters have a variety of jobs (Junior Firefighter, Firefighter, Senior Firefighter, Platoon Commander, Station Commander, District Commander, Chief Fire Officer), duties (fires, traffic collisions, emergency resuscitation, training and drills, equipment maintenance, etc.), and functions (emergency response, public safety, education and training) to perform that vary greatly by country and change over the time-course of their careers (e.g., from junior firefighters to ranked officers) [2,7,8,9,10]. Reductions in pulmonary function and increased pulmonary symptoms have been seen in firefighters following fire and fume exposure while on duty, and these symptoms become more severe with increased exposure [11,12,13,14]. In addition, the long-term deleterious effects of chronic smoke inhalation, commonly seen in firefighters, directly raises the risk of pulmonary disorders in this population [6,15].

Research has shown that firefighters had decreased pulmonary functions and capacities due to frequent occupational smoke inhalation and, consequently, presented with several pulmonary symptoms, which included chest tightness, shortness of breath, coughing and wheezing [15,16]. According to studies on wildland firefighters, the fire season was associated with increased occupational stress and a decrease in pulmonary health with worsening pulmonary symptoms [17,18]. Furthermore, hazardous occupational exposures are instrumental in the development of pulmonary disorders [19]. These exposures may lead to or worsen chronic pulmonary disorders such as asthma, emphysema, chronic bronchitis, bronchiolitis, bronchiectasis, interstitial pulmonary fibrosis, and sarcoidosis [19]. Therefore, entering the firefighting occupation with a pre-existing pulmonary disease, such as asthma, often leads to disease exacerbation [12,20]. Therefore, being in a healthy physical state, prior to being admitted into the firefighting service, is also of utmost importance. Firefighters who are overweight or obese were more likely to sustain injury, succumb to cardiac episodes and/or develop chronic pulmonary disorders [21].

Research in the United States and Europe demonstrated a substantial reduction in pulmonary function in firefighters [13]. Betchley et al. [22] observed a reduction of 65 mL in forced vital capacity (FVC) and a reduction of 150 mL in forced expiratory volume in one second (FEV_1.0_) throughout the firefighters’ careers and as they aged among firefighters in the United States. Similarly, Jacquin et al. [23] reported a reduction of 59 mL in FVC and a decrease of 53 mL in FEV_1.0_ among French firefighters throughout their careers and as they aged. In addition, studies have found that a decrease in forced mid-maximal expiratory flow (FEF_25–75_) suggested the presence of obstructive airflow in the small airways [22,23]. Liu et al. [24] found that Californian firefighters had significant decreases in FVC by 90 mL, FEV_1.0_ by 150 mL, and FEF_25–75_ by 440 mL/s, across different seasons. The findings aligned with the research conducted by Betchley et al. [22] in wildland firefighters, which indicated reductions of 33 mL in FVC, 104 mL in FEV_1.0_, and 275 mL/s in FEF_25–75_. Moreover, in a study on South Korea firefighters’ pulmonary function progressively declined among firefighters throughout their careers [14]. Similarly, in Australia, 10% of metropolitan firefighters were found to have persistent pulmonary difficulties [25].

There have not been any studies conducted on the pulmonary functions and capacities of firefighters in South Africa [3,5]. The conditions in South Africa present a particularly concerning environment, with firefighters exposed to copious amounts of fire smoke, particularly in informal settlements, which have the added burden of releasing exorbitant amounts of toxic chemical fumes [26,27]. Due to the nature of these fires, firefighters may be predisposed to chronic lung conditions and decreased functioning and capacities, significantly reducing their occupational performance, placing them at risk while on duty [26,27]. Therefore, this study aimed to determine the prevalence of pulmonary symptoms and disorders among full-time firefighters in the City of Cape Town Fire and Rescue Service and to explore associations with sociodemographic, physical and behavioural risk factors.

## 2. Materials and Methods

### 2.1. Study Design, Participant Preparation, Selection and Screening

This study utilised a quantitative cross-sectional, descriptive and correlational design conveniently recruiting a total of 278 full-time firefighters, comprising males and females aged 18 years and older in the City of Cape Town Fire and Rescue Service (CoCTFRS). Using Slovin’s formula [n = N/(1 + N e^2^) = n = 900/(1 + 900e^2^) = 277], this study met the minimum sample required for the population of firefighters in the CoCTFRS. The mean age of the firefighters was 39.65 ± 10.18 years. The majority of firefighters were male (86%), with a mean age of 39.86 ± 10.52 years, and females were 38.41 ± 7.73 years. The mean heights of the male and female firefighters were 173.80 ± 6.73 and 161.53 ± 5.91 cm, respectively. Male firefighters had a mean body mass of 85.88 ± 15.35 kg and females 80.56 ± 20.00 kg.

**Figure 1:**
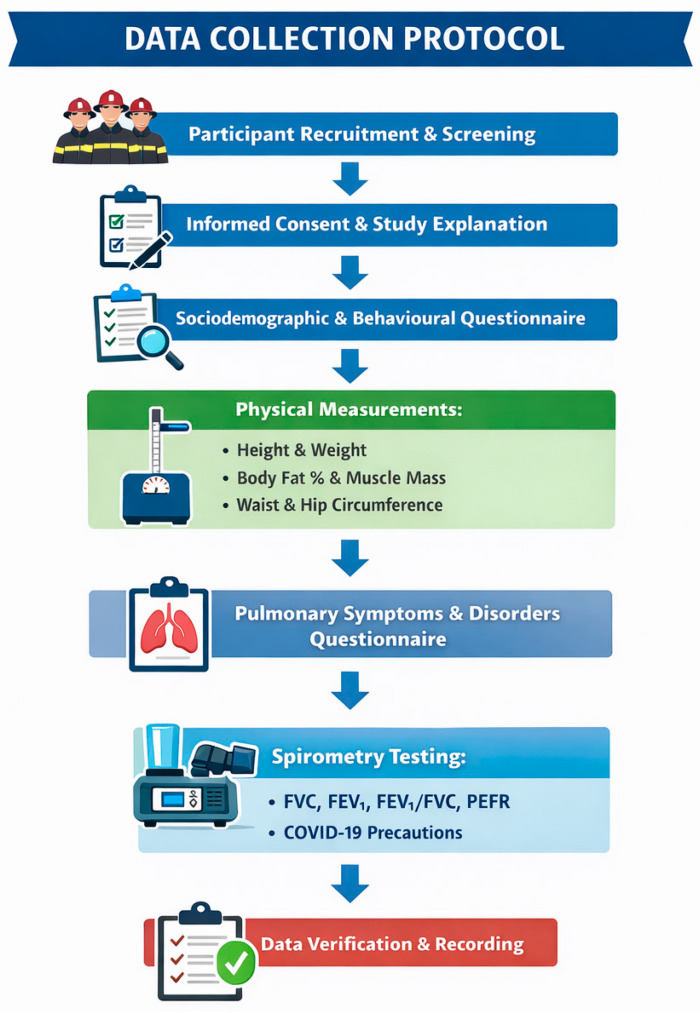
Data collection procedure.

Information and pre-test instructions were given to the station commanders who forwarded the information to the firefighters. Pre-test instructions and testing procedures were then explained to the firefighters again on the day of testing. The duration of each assessment was scheduled for 30 min per firefighter, while in some instances the time was extended due to technical issues. Due to firefighting being an emergency service occupation and in the case of a call during testing, the firefighters who missed the testing window were rescheduled for their next shift on duty. Sociodemographic characteristics (age, sex, rank, marital status, educational level and medical history) and behavioural risk factors (years of service, alcohol consumption, smoking behaviour and physical activity (while off duty)) were recorded via a validated researcher-generated questionnaire, which the firefighters completed electronically. Ethical clearance to conduct this study was obtained from the Biomedical Research Ethics Committee (BMREC) at the University of the Western Cape (Ethical clearance number: BM23/3/13). In addition, permission to test the firefighters was obtained from the City of Cape Town, as well as the Chief Fire Officer of the CoCTFRS. Information about this study was provided to potential participants, and then written consent was obtained. The risks of the spirometry test, such as the potential for infection from the equipment, were explained to participants, particularly for those with symptoms of colds, flu, or tuberculosis. Participation in this study was voluntary with the option of withdrawing at any stage without any negative consequences. This study included full-time male and female firefighters permanently employed by the CoCTFRS who were between 18 and 65 years of age. Administrative staff who were not active firefighters, volunteer firefighters, and individuals employed on a short-term or seasonal contract basis were excluded from participation. In addition, firefighters who presented with contraindications to spirometry testing, as outlined by the American Thoracic Society (ATS), the European Respiratory Society (ERS), and the South African Thoracic Society (SATS), were not eligible to participate in this study. The entire data collection protocol is explained by Figure 1.

### 2.2. Physical Measurements

#### 2.2.1. Stature

Stature was measured using a stadiometer (Seca model 700, Gmbh & Co., Hamburg, Germany) with the firefighter standing barefoot on a level floor with the heels together and the heels, buttocks and upper back touching the stadiometer rod [28]. The participant’s head was positioned in the Frankfort plane, which is a horizontal plane aligned by placing the tips of the tester’s thumbs on the orbitale and the tips of the index fingers on the trigion [28]. The participant was instructed to inhale and hold their breath, while the stadiometer rod was gradually lowered onto the top of their head (vertex), exerting maximum pressure on the hair. Height was recorded to the nearest 0.1 cm, at least twice [29,30].

#### 2.2.2. Body Mass, Body Fat Percentage and Muscle Mass

A bioelectrical impedance (BIA) analyser (Tanita BC-1000plus, Tokyo, Japan) was used to obtain body composition data, which will include body mass, body fat percentage (BF%) and muscle mass (MM). Body mass was privately assessed while participants wore minimal clothing, such as shorts for males and shorts with a light top or swimsuit for females. For the BIA assessment, firefighters were requested to wear minimal clothing, to stand upright, barefoot and stationary on the scale. Obesity was assessed using body mass index (BMI), which was determined by dividing the participant’s body mass in kilograms by their height in metres squared. BMI is represented in kilograms per square metre (kg·m^2^).

#### 2.2.3. Waist and Hip Circumferences

The waist circumference (WC) was measured horizontally above the umbilicus and below the xiphoid process, which is the narrowest section of the torso located between the lower costal border (10th rib) and the top of the iliac crest. The measurement was taken perpendicular to the long axis of the trunk. The cross-hand technique was employed to measure the circumferences of all objects [28]. The measuring tape was aligned perpendicular to the body segments being measured, with the hook tab grasped in the right hand and the stub held in the left hand. The left hand was used to adjust the tape to the appropriate position and subsequently slid beneath the casing to seize the counterfoil once more. The subject utilised their middle fingers on both hands to accurately position the tape measure at the designated reference point and align it in a manner that allowed for easy reading of the zero mark, ensuring that the tester’s eyes were level with the tape measure [28]. The hip circumference was measured at the point on the buttocks that sticks out the most, which is parallel to the level of the symphysis pubis and represents the widest region of the hips. Waist and hip circumference were measured with an accuracy of 0.1 cm at the end of a regular exhale [30,31]. The measurements were conducted on two occasions and the mean value was recorded as the final measurement, as long as the difference between the two measurements was less than 3 mm [30,31]. The waist-to-hip ratio (WHR) was determined by dividing the waist circumference (WC) by the hip circumference [32]. This measurement indicated the distribution of fat in the participant’s body, namely whether it is concentrated in the core (android) or peripheral (gynocoidal) areas.

#### 2.2.4. Behavioural Measurements

Smoking status was determined if firefighters were current smokers or had quit within the last 6 months. Physical activity was determined according to the ACSM guidelines, meeting the minimum standards of physical fitness, which was exercising at least 30 min on at least three days a week at a moderate intensity, accumulating 150 min per week [28]. Firefighters were classified as insufficiently active due to not meeting the minimum of 150 min of moderate-intensity activity or not exercising at least 30 min on at least 3 days per week. Exceeding the recommended 150–300 min of moderate-intensity or 75–150 min of vigorous-intensity aerobic activity per week [28].

#### 2.2.5. Pulmonary Symptoms and Pulmonary Disorders

A previously validated questionnaire, the Pulmonary Function Questionnaire (LFQ), was utilised to record pulmonary symptoms of firefighters [15]. Firefighters were required to answer questions related to their breathing. The first question focused on the firefighters experiencing wheezing or whistling in the chest at any time in the past year or 12 months. The second question focused on the firefighters experiencing attacks of shortness of breath with wheezing. The third question asked firefighters to rate how limited their activities and duties were due to shortness of breath, wheezing, coughing and/or chest tightness in the four weeks prior to the study. Pulmonary disorders were recorded via a validated researcher-generated questionnaire, which the firefighters completed electronically. Firefighters had to state any previous or current pulmonary disorders. The researcher-generated questionnaire included questions on their demographic characteristics, including the fire station they work at, their sex, date of birth, core job description, years of experience and marital status. The questionnaire included sections on their current medical conditions, smoking status and physical activity levels (international physical activity questionnaire).

#### 2.2.6. Spirometry Testing

Pulmonary functions and capacities were obtained using the nSpire KoKo^®^ PFT system (nSpire Health, Inc., Longmont, CO, USA [Model 313106, Serial 1523K3316]). Contraindications to spirometry were explained to the firefighters in accordance with the guidelines of the American Thoracic Society (ATS), the European Respiratory Society and the South African Thoracic Society. The KoKo spirometer was calibrated at each fire station before measurement. The environment was monitored, and ambient conditions was checked to ensure that they were within accepted ranges. The best test from an acceptable curve that had the highest sum of forced vital capacity (FVC) and forced expiratory volume in one second (FEV_1.0_) from the expiratory manoeuvre was used for the research. In accordance with the World Health Organization (WHO) [33] COVID-19 safety guidelines, the following precautions were taken: (1) personal protective equipment was worn by the researchers at all times, which includes the use of disposable gloves covering the hands, disposed of after each participant; (2) hand hygiene was ensured through hand-washing before and after each participant was tested, as well as hand sanitisation with alcohol-based sanitiser, before and after each participant was tested; and (3) equipment hygiene was ensured by using disposable mouthpieces in the spirometer for each participant and through disinfecting the equipment before and after each use. Predicted percentage was calculated using the National Health and Nutrition Examination Survey (NHANES) [34,35] and ATS [36] guidelines. In addition, lung function was assessed using standardised spirometry procedures conducted in accordance with ATS guidelines [34,35,36], with multiple forced expiratory manoeuvres performed to obtain the highest acceptable and reproducible values for key indices such as forced vital capacity (FVC) and forced expiratory volume in one second (FEV_1_). Observed spirometric values were then compared with population-based reference equations that account for age, sex, height, and race/ethnicity to generate predicted values [34,35]. Percent predicted was calculated as (observed value ÷ predicted value) × 100, while lung function abnormality was further evaluated using z-scores, computed as (observed − predicted) ÷ standard deviation of the reference population [34,35]. The lower limit of normal (LLN) was defined as the 5th percentile of the reference distribution (z = −1.64), allowing for classification of ventilatory impairment and severity based on statistically derived thresholds rather than fixed cut-off values [34,35,36].

### 2.3. Instrument and Tester Reliability and Validity

The research spirometry equipment used for data collection was serviced by the department and calibrated at each fire station by the researcher. In order to ensure intra-tester reliability and validity, only one tester was used in the study [31]. A BIA Tenita scale of excellent quality was utilised to guarantee the accuracy and credibility of the results. The BIA scale is renowned for its accuracy and has a margin of error of 3%, which is deemed acceptable for clinical and research applications. In addition, WC and hip circumference were used to evaluate the distribution of body fat. These assessments are robust and dependable indicators of central adiposity, a crucial component in pulmonary function and general health. In order to guarantee the accuracy of these measures, standardised protocols were adhered to, and the tester underwent appropriate training. This methodology guaranteed a strong level of agreement and consistency leading to reliable and accurate results.

### 2.4. Statistical Analysis

The data were recorded using the double-entry method in a Microsoft Office Excel spreadsheet and subsequently underwent mistake correction. Subsequently, the data were transferred to the Statistical Package for the Social Sciences (SPSS) version 29 for the purpose of conducting data analysis. The researcher kept all electronic backup copies of the data on a computer, within password-protected folders, and regulated access to them. Every participant was assigned an alphanumeric code to safeguard their anonymity while gathering and recording the data onto the spreadsheets (Microsoft Excel and SPSS). Descriptive statistical analysis (mean, standard deviation and frequencies) and inferential statistics (Kruskal–Wallis H, Chi-squared test and Pearson’s correlation coefficient) were used for continuous variables, such as age, height, weight, BF%, pulmonary functions and capacities. The Kruskal–Wallis H test was used to test for differences in various pulmonary functions and capacities based on physical characteristics (BMI, BF%, WC and WHR), sociodemographic characteristics (rank, marital status, educational level and medical history), and behavioural risk factors (smoking status, physical activity and alcohol consumption). A post hoc Bonferroni correction was applied to the findings to minimise type I error. The Chi-squared test was used for determining the association between categorical variables, such as age-groups and sex, and pulmonary functions and capacities. Pearson’s correlation was utilised to determine the relationships between pulmonary functions and capacities and physical and sociodemographic characteristics, as well as between the behavioural risk factors of the firefighters. In addition, univariable and multivariable linear regressions were conducted to determine the association between lung function and capacities and physical and behavioural characteristics. Multivariable models were adjusted for age and height, as these factors have been shown to be significantly associated with lung function and capacities [16,25]. A *p* value of less than 0.05 was used to indicate statistical significance. 

## 3. Results

**Figure 2:**
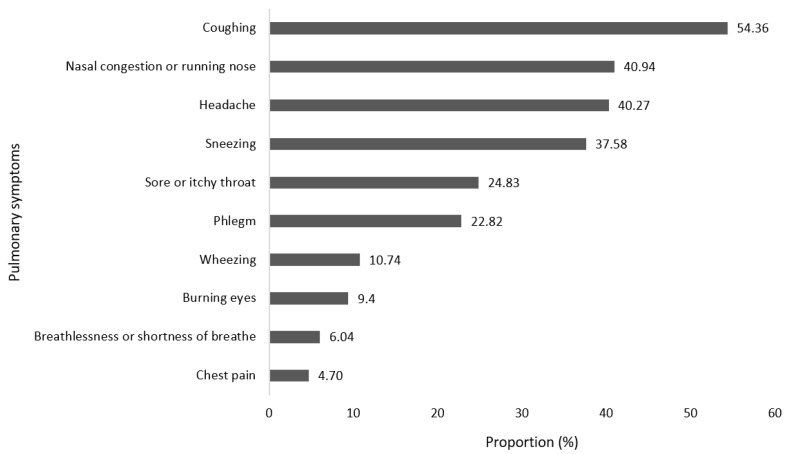
Pulmonary symptoms in firefighters.

More than half of the firefighters (53.60%) presented with pulmonary symptoms in the four weeks preceding their participation in the study (Figure 2). Among these firefighters, 54.36% presented with coughing, 40.94% with nasal congestion or rhinorrhoea, 40.27% presented with headache, 24.83% had a sore or itchy throat, 22.82% had phlegm, 10.74% had wheezing, 9.4% had burning eyes, 6.04% had breathlessness or shortness of breath and 4.70% had chest pain. In addition, 30.1% of firefighters reported having two or more symptoms, which may have presented with more than one symptom, indicating lower lung function.

Table 1 delineates the pulmonary functions and capacities of firefighters based on physical activity and years of service. In terms of PA, insufficiently active, sufficiently active and highly active firefighters had slight or mild restriction, and one sufficiently active firefighter had severe restriction. Regarding years of service, firefighters with 20 or more years of service presented with pulmonary restriction. Obstructive pulmonary patterns were more commonly found among firefighters who were sufficiently active or highly active. This was also related to years of service, where firefighters with longer service (≥20 years) presented with pulmonary obstruction. The results for FEF_25–75_ showed that mid-airway obstruction tended to be affected by all categories of PA and years of service.

Table 2 describes the pulmonary functions and capacities of firefighters based on sex, age-group and smoking status. A total of 97.12% of firefighters presented with normal forced expiration or no obstructive patterns (FEV_1.0_ > 80%). Borderline obstruction (FEV_1.0_ = 80%) was found in 0.36% of firefighters, mild pulmonary obstruction (FEV_1.0_ = 60–79%) in 2.16%, moderate obstruction (FEV_1.0_ = 40–59%) in 0.36%, and none had severe pulmonary obstruction (FEV_1.0_ < 40%). A total of 63.31% of firefighters presented with normal forced mid-maximal expiratory flow (FEF_25–75_ > 80%). Borderline midway obstruction (FEF_25–75_ = 80%) was found in 23.38%, mild obstruction (FEF_25–75_ = 60–79%) in 10.43%, moderate obstruction (FEF_25–75_ = 40–59%) in 1.08% and severe obstruction (FEF_25–75_ < 40%) in 1.80%. Peak expiratory flow rate (PEFR) was normal (PEFR ≥ 70%) for the majority of firefighters (95.68%), whereas 4.32% presented with reduced PEFR (PEFR < 70%). For both sexes, the majority of firefighters had no pulmonary restriction. This was supported by the results for FEV_1.0_, which also showed that the majority of firefighters had no pulmonary obstruction. The results for FEF_25–75_ showed that mid-airway obstruction tended to be age-related but not influenced by smoking behaviour.

Table 3 illustrates the differences in pulmonary functions and capacities of firefighters based on age-group in firefighters. There was a significant difference between male and female firefighters for pulmonary functions and capacities for FEV_1.0_/FVC (ratio) (*p* < 0.001). There were significant differences in FVC (L·min^−1^) (*p* <0.001) and FEV_1.0_ (L·min^−1^) based on age-group (*p* <0.001).

Table 4 describes the differences in pulmonary functions and capacities of firefighters based on smoking status. The results indicated that there was a significant difference between smokers and non-smokers in FEV_1.0_/FVC (ratio) (*p* < 0.001).

Table 5 describes the association between the pulmonary functions and capacities and the smoking behaviour of firefighters. There were statistically significant associations between FVC and the number of cigarettes smoked (χ^2^ = 2002.8, *p* < 0.01) and between PEFR and the number of cigarettes smoked (χ^2^ = 2466.21, *p* < 0.05).

Table 6 describes the differences between pulmonary functions and capacities of firefighters based on BMI categories. There was a significant difference in FVC between BMI categories (*p* < 0.001). In addition, there was a significant difference in FEV_1.0_ between BMI categories (*p* < 0.001).

Table 7 reports the differences in pulmonary functions and capacities of firefighters based on waist-to-hip ratio. There were significant differences in FVC (*p* < 0.001), FEV_1.0_ (*p* < 0.001), FEV_1.0_/FVC (*p* < 0.001), FEF_25–75_ (*p* < 0.001)_,_ and PEFR (*p* < 0.001) in firefighters.

Table 8 delineates the relationship between the pulmonary functions and capacities and the physical characteristics of firefighters. There were statistically significant and moderate positive correlations between MM and FVC (r = 0.51, *p* < 0.01), MM and FEV_1.0_ (r = 0.49, *p* < 0.01), and MM and PEFR (r = 0.47, *p* < 0.01). There were statically significant and moderate negative correlations between BF% and FVC (r = −0.47, *p* < 0.01) and between BF% and FEV_1.0_ (r = −0.48, *p* < 0.01). A statistically significant and weak positive correlation was found between MM and FEF_25–75_ (r = 0.26, *p* < 0.01). There were statistically significant and weak negative correlations between BF% and FEF_25–75_ (r = −0.23, *p* < 0.01), BMI and FVC (r = −0.36, *p* < 0.01), BF% and PEFR (r = −0.30, *p* < 0.01), and BMI and FEV_1.0_ (r = −0.37, *p* < 0.01). There were statistically significant and very weak negative correlations between MM and FEV_1.0_/FVC (r = −0.13, *p* < 0.05), BMI and FEF_25–75_ (r = −0.13, *p* < 0.05), and BMI and PEFR (r = −0.15, *p* < 0.05).

Table 9 shows the relationship between the pulmonary functions and capacities and the behavioural risk factors of firefighters. A statistically significant and very weak positive correlation was found between alcohol consumption and years of service (r = 0.15, *p* < 0.01). Statistically significant and very weak negative correlations were found between years of service and PA (r = −021, *p* < 0.01) and between alcohol consumption and PA (r = −0.22, *p* < 0.05). There was a statistically significant and weak positive correlation between PA and FVC (r = 0.20, *p* < 0.01). There were statistically significant and very weak positive correlations between PA and FEV_1.0_ (r = 0.19, *p* < 0.01) and between PA and PEFR (r = 0.14, *p* < 0.05). There were statistically significant and very weak negative correlations between years of service and FVC (r = −0.19, *p* < 0.01), years of service and FEV_1.0_ (r = −0.23, *p* < 0.01), and years of service and FEF_25–75_ (r = −0.13, *p* < 0.05). There was a statistically significant and weak positive correlation between PA and FVC (r = 0.20, *p* < 0.01). There were statistically significant and very weak positive correlations between PA and FEV_1.0_ (r = 0.19, *p* < 0.01) and between PA and PEFR (r = 0.14, *p* < 0.05). There were statistically significant and very weak negative correlations between years of service and FVC (r = −0.19, *p* < 0.01), years of service and FEV_1.0_ (r = −0.23, *p* < 0.01), and years of service and FEF_25–75_ (r = −0.13, *p* < 0.05). Average alcohol units were significantly correlated with FVC (r = −0.147, *p* = 0.048) and FEV_1_ (r = −0.166, *p* = 0.025).

Table 10 delineates the linear association between pulmonary functions and capacities, physical characteristics, and behavioural risk factors of firefighters. Univariable models indicated that age, years of service, BF%, MM and physical activity was significantly associated with most measures of lung functions and capacities. In the multivariable models after adjustment for age and height, age remained significantly associated with FVC (*p* < 0.001), FEV_1_ (*p* = 0.001) and FEF_25–75_ (*p* = 0.003). Years of experience only remained associated with FEF_25–75_. BMI remained significantly associated with FVC (*p* = 0.014) and FEV_1_ (*p* < 0.001). BF% remained significantly associated with FVC (*p* < 0.001), FEV_1_ (*p* < 0.001) and PEFR (*p* = 0.002). MM remained significantly associated with FVC (*p* < 0.001) and PEFR (*p* = 0.004). Physical activity remained significantly associated with FVC (*p* = 0.022).

## 4. Discussion

During the present investigation, the majority of firefighters presented with coughing, followed by nasal congestion or rhinorrhoea, headaches, sore or itchy throat and phlegm. The symptoms described are consistent with the findings reported by Gianniou et al. [6], where pulmonary symptoms such as wheezing (33%), cough (71%), chest tightness (18%), sneezing (50%), and expectoration (33%) were substantially more prevalent following firefighting activities compared to off-season periods. The most reported symptoms were sputum cough (33.3%), dyspnoea (32.2%), wheezing (20%), and coughing (14.4%). This is further corroborated by Agin et al. [37] who found that 98% of firefighters encountered pulmonary symptoms related to the upper pulmonary tract, including the generation of sputum and persistent coughing. Chean et al. [38] found that firefighters and traffic officer had significantly higher pulmonary symptom scores compared to the general population, regardless of behavioural risk factors.

The results of the current study showed that 97.84% of firefighters exhibited normal pulmonary tissue compliance. Borderline restriction was observed in 0.72% of firefighters, with an FVC of 80%. Restriction was found in 1.08% of firefighters, while severe restriction was detected in 0.36%. Isara and Egbagbe [39] conducted a study in Nigeria and discovered that 52.5% of firefighters had restrictive patterns. Ramos and Minghelli [1] conducted a study in Portugal and found that 68% of wildfire firefighters exhibited pulmonary restrictive patterns.

The current study revealed significant differences in FEV_1.0_/FVC between firefighters who smoke and those who do not smoke. In addition, statistically significant associations were found between FVC and PEFR and the number of cigarettes smoked. These findings are consistent with prior research that emphasised the harmful effect of smoking on pulmonary function [16]. Previous studies conducted on firefighters who were exposed to dust during the World Trade Centre catastrophe revealed that firefighters who smoke had significantly poorer pulmonary function in comparison to those who have never smoked and those who used to smoke, with the latter group experiencing moderate decreases [6,40]. Firefighters who have smoked in the past suffered a considerable decline in pulmonary function compared to those who have never smoked [25]. Previous research has also found a greater occurrence and seriousness of obstructive airway disease in firefighters who smoke, emphasising a particular harmful outcome of smoking [13]. Moreover, smoking triggers chronic inflammation and damage to the pulmonary system, making smokers more vulnerable to additional injury caused by environmental exposures [25]. The increased susceptibility reported in persons who smoke or smoked in the past may explain the more significant decline in pulmonary function compared to those who never smoked [25,40]. Engaging in smoking and being exposed to dangerous occupational particles, such as dust and chemicals found in firefighting, are more likely to exacerbate the decline in pulmonary function [6].

The present study found significant differences in PA categories for FVC, FEV_1.0_, and PEFR. In addition, there were significant relationships between PA and FVC, FEV_1.0_, and PEFR, which remained after adjustment for confounders. Multiple studies have demonstrated that engaging in more physical activity decreases the probability of getting chronic obstructive pulmonary disease (COPD) and improves pulmonary function [1,12]. A study by Babu et al. [41] emphasises the significance of integrating various types of physical activity in order to optimise the advantages for pulmonary health. The findings of our study are consistent with this, demonstrating significant correlations between PA and enhanced pulmonary function. Firefighters should maintain their pulmonary health through exercise and behaviours conducive to enhanced lung function, directly improving their occupational functioning and reduce the incidence of work-related lung disease or on-duty fatalities [18,19,42].

There were significant relationships found between age and FVC and FEV_1.0,_ which remained significant after adjustment for confounders. The ageing process entails several alterations in the pulmonary system, including adjustments in the dimensions of the thoracic cavity, limitations in pulmonary volumes, and changes in breathing muscles [13]. According to Kim et al. [43], ageing increased firefighters’ susceptibility to developing pulmonary disorders and experiencing a decrease in pulmonary function. Similarly, Babu et al. [41] found that firefighters face a higher risk of developing obstructive airway disease as they get older. Mathias et al. [13] reported that firefighters experienced pulmonary function decline that was two to four times larger than that observed in the general population during the same time frame. Aldrich et al. [40] documented a 10% reduction in FEV_1.0_ among firefighters within the initial year after being exposed to World Trade Centre dust. Slattery et al. [16] discovered a significant relationship between age and pulmonary function, where younger firefighters exhibited higher FEV_1.0_ and FVC scores that decreased as they aged. The correlations highlight the influence of ageing on the decrease in pulmonary function among firefighters [13,41]. Older firefighters are more prone to pulmonary disorders and experience more significant deterioration in pulmonary function [25]. Therefore, specific behavioural interventions should be implemented to reduce age-related pulmonary function decline in this population [16,43].

We found statistically significant differences in years-of-service categories of firefighters for FVC, FEV_1.0_, FEF_25–75_, and PEFR. There were statistically significant and very weak negative correlations between years of service and FVC, FEV_1.0_, and FEF_25–75_. An inverse relationship exists between FEV_1.0_ and years of service, indicating that the firefighting occupation has a cumulative detrimental effect on pulmonary health. These findings are consistent with previous research conducted by Choi et al. [14], where firefighters who have been in service for more than five years face significant reductions in FEV_1.0_, FVC, PEFR, FEV_1.0_/FVC, in comparison to those with shorter lengths of service. The study highlighted the necessity for focused behavioural interventions to maintain pulmonary health in the firefighting population [14]. In the current study, there were significant differences in FVC among individuals with less than 5 years of duty compared to those with 25–30 years of service. The findings aligned with prior studies conducted by Choi et al. [14] and Kim et al. [12], which concluded that firefighters with over five years of work experience demonstrated significant decline in pulmonary function compared to individuals with shorter periods of service. The findings highlight the importance of implementing specific measures, such as consistent use of a self-contained breathing apparatus, post-fire decontamination procedures, routine respiratory health surveillance, education and behavioural interventions and wellness programmes to reduce the long-term dangers to pulmonary health from extended exposure.

The present study found statistically significant relationships between BMI and FVC, as well as BMI and FEV_1.0_, which remained significant after adjustment for confounders. Isara and Egbagbe [39] found substantial negative correlations between BMI and pulmonary function, specifically in FEV_1.0_, FVC, the FEV_1.0_/FVC ratio, and PEFR. The study suggests that BMI has a major impact on pulmonary performance in firefighters. Similarly, Dunga et al. [44] and Wannamethee et al. [45] reported significant correlations between greater BMI and poorer FEV_1.0_/FVC ratios. Research has demonstrated that obesity can imitate symptoms of asthma by reducing the capacity of the pulmonary system and raising the resistance of the airways [44,46,47]. The findings of the present study are consistent with these conclusions, indicating that higher BMI is linked to decreased FVC, FEV_1.0_, and PEFR in firefighters. This relationship is further supported by the high frequency of overweight among firefighters, which leads to a decline in pulmonary function in the current study. In adult populations without asthma, there is an inverse relationship between BMI and FVC, meaning that as BMI increases, FVC decreases [12,48]. This suggests that BMI has an independent impact on pulmonary function, separate from the effects of smoke inhalation. The findings of the present investigation support the notion that weight gain hastens the decline of pulmonary function, a pattern that has been documented in other long-term studies [49]. Independently, weight gain, and obesity in particular, has been associated with reduced occupational health and performance, due to excessive weight [3,5,50,51]. The current results indicated that not only does obesity reduce work performance due to increased adiposity, it may reduce lung function, reducing oxygen transportation, subsequently leading firefighters to reach volitional fatigue earlier [52,53].

The current study found significant relationships between pulmonary function and BF% and BMI, particularly in terms of FVC, FEV_1.0_, FEF_25–75_, and PEFR, which remained significant after adjustment for confounders. Obesity decreased pulmonary capacities and increased airway resistance, which imitated asthma symptoms and led to a decline in pulmonary function [46]. Previous research demonstrated that increased body weight, WC and WHR were strong indicators of deleterious pulmonary function [44,54], which was also reported in the current study. In addition, the present study demonstrated significant relationships between MM and FVC, FEV_1.0_, and PEFR, which remained significant after adjustment for confounders. The findings of the current study align with previous studies indicating that an increase in MM positively impacts pulmonary function, but a higher proportion of BF% is correlated with a decline in pulmonary function [12,55]. This emphasised the importance of implementing therapies that reduce body fat and increase MM in firefighters to maintain pulmonary function [12]. A study that employed the skeletal muscle mass index discovered a connection between a reduction in MM and a fall in FVC, FEV_1.0_, and PEFR [12,55]. Dunga et al. [44] discovered that there was a decline in pulmonary function when BF% increased. There is a debate on whether a direct correlation exists between obesity and asthma or whether obesity leads to asthma-like symptoms, which requires further investigation [44,45,46,56].

### Strengths and Limitations

This study offers unique insights into the pulmonary function and capacity of firefighters in South Africa, specifically in the City of Cape Town Fire and Rescue Service, an area that has received limited research attention. The relatively large sample size provides strong statistical power for the analysis that was conducted. The utilisation of convenience sampling negatively impacted the external validity of this study. In addition, this study had a limited number of female firefighters, which could impact the generalisability to the female firefighter population. Furthermore, this study did not examine the degree of smoke inhalation experienced by firefighters in their work environment, which is a key determinant in the development of pulmonary disorders and symptoms. This study’s cross-sectional design restricts its capacity to show a causal relationship between occupational exposures and pulmonary diseases. Lastly, hydration status was not controlled for and may have influenced the BIA results.

## 5. Conclusions

The present study offers valuable insights on the pulmonary function and capacity of firefighters in South Africa, specifically in the City of Cape Town Fire and Rescue Service, which has received limited research attention. This study identified the presence of both restrictive and obstructive pulmonary disorders among firefighters and highlighted the potential impact of body composition, particularly BF% and MM, on pulmonary function. These findings emphasise the importance of regular pulmonary function testing and ongoing monitoring of BMI. Furthermore, the observed relationship between body composition, behavioural risk factors, and pulmonary function underscores the need for tailored health management strategies. Regular exercise plays a critical role in mitigating the adverse effects of prolonged occupational exposure on pulmonary health. Therefore, comprehensive health programmes that promote physical activity, reduce body fat percentage, enhance muscle mass, and address negative health behaviours are essential.

### Recommendations for Future Research

Future research should aim to include a larger and more balanced sample of male and female firefighters to improve the generalisability of findings and better understand gender-specific pulmonary responses. Studies should incorporate objective measures of smoke inhalation and other occupational exposures to clarify their impact on pulmonary function, while longitudinal designs could help establish causal relationships between occupational hazards, body composition, lifestyle factors, and pulmonary health. Lastly, research evaluating targeted interventions, such as structured fitness programs, nutritional strategies, and enhanced protective equipment, would provide practical guidance for mitigating pulmonary function decline.

## Figures and Tables

**Table 1:** Pulmonary functions and capacities of firefighters based on physical activity and years of service.

Pulmonary Functions and Capacities	Physical Activity (While Off Duty)								Years of Service													
	Sedentary (n = 60)		Insufficiently Active (n = 20)		Sufficiently Active (n = 24)		Highly Active (n = 174)		<5 (n = 47)		5–9 (n = 53)		10–14 (n = 40)		15–19 (n = 50)		20–24 (n = 28)		25–30 (n = 31)		>30 (n = 29)	
	n	%	n	%	n	%	n	%	n	%	n	%	n	%	n	%	n	%	n	%	n	%
Forced vital capacity																						
Normal	60	100	19	95.00	22	91.67	171	98.28	47	100	53	100	40	100	50	100	25	89.29	29	93.55	28	96.55
Borderline restriction	0	0.00	0	0.00	0	0.00	2	1.15	0	0.00	0	0.00	0	0.00	0	0.00	0	0.00	1	3.23	1	3.45
Slight or mild restriction	0	0.00	1	5.00	1	4.17	1	0.58	0	0.00	0	0.00	0	0.00	0	0.00	2	7.14	1	3.23	0	0.00
Moderate restriction	0	0.00	0	0.00	0	0.00	0	0.00	0	0.00	0	0.00	0	0.00	0	0.00	0	0.00	0	0.00	0	0.00
Severe restriction	0	0.00	0	0.00	1	4.17	0	0.00	0	0.00	0	0.00	0	0.00	0	0.00	1	3.57	0	0.00	0	0.00
Forced expiratory volume in one second																						
Normal	58	96.67	19	95.00	21	87.50	172	98.85	47	100	52	98.11	40	100	48	96.00	26	92.86	30	96.77	27	93.10
Borderline obstruction	1	1.67	0	0.00	0	0.00	0	0.00	0	0.00	0	0.00	0	0.00	0	0.00	0	0.00	0	0.00	1	3.45
Slight or mild obstruction	1	1.67	1	5.00	2	8.33	2	1.15	0	0.00	1	1.89	0	0.00	2	4.00	1	3.57	1	3.23	1	3.45
Moderate obstruction	0	0.00	0	0.00	1	4.17	0	0.00	0	0.00	0	0.00	0	0.00	0	0.00	1	3.57	0	0.00	0	0.00
Severe obstruction	0	0.00	0	0.00	0	0.00	0	0.00	0	0.00	0	0.00	0	0.00	0	0.00	0	0	0	0.00	0	0.00
Forced mid-maximal expiratory flow																						
Normal	36	60.00	13	65.00	13	54.17	114	65.52	29	61.70	30	56.60	26	65.00	28	56.00	23	82.14	19	61.29	21	72.41
Borderline obstruction	1	1.67	0	0.00	2	8.33	2	1.15	1	2.13	0	0.00	1	2.50	2	4.00	1	3.57	0	0.00	0	0.00
Slight or mild obstruction	17	28.33	5	25.00	5	20.83	38	21.84	13	27.66	12	22.64	12	30.00	14	28.00	2	7.14	8	25.81	4	13.79
Moderate obstruction	5	8.33	2	10.00	3	12.50	19	10.92	4	8.51	11	20.76	1	2.50	5	10.00	2	7.14	3	9.68	3	10.35
Severe obstruction	1	1.67	0	0.00	1	4.17	1	0.58	0	0.00	0	0.00	0	0.00	1	2.00	0	0.00	1	3.23	1	3.45
Peak expiratory flow rate																						
Normal	56	93.33	19	95.00	22	91.67	168	96.55	44	93.62	52	98.11	38	95.00	49	98.00	27	96.43	27	87.10	28	96.55
Borderline obstruction	1	1.67	0	0.00	0	0.00	0	0.00	0	0.00	0	0.00	0	0.00	0	0.00	0	0.00	1	3.23	0	0.00
Obstruction	3	5.00	1	5.00	2	8.33	6	3.45	3	6.38	1	1.89	2	5.00	1	2.00	1	3.57	3	9.68	1	3.45

Note: FVC: normal > 80% of predicted FVC; borderline restriction = 80% of predicted FVC; slight or mild restriction = 60–79% of predicted FVC; moderate restriction = 50–59% of predicted FVC; severe restriction < 50% of predicted FVC. FEV_1.0_: normal > 80% of predicted FEV_1.0_; borderline obstruction = 80% of predicted FEV_1.0_; slight or mild obstruction = 60–79% of predicted FEV_1.0_; moderate obstruction = 40–59% of predicted FEV_1.0_; severe obstruction < 40% of predicted FEV_1.0_. PEFR: normal ≥ 70% of predicted PEFR; reduced <70% of predicted PEFR.

**Table 2:** Pulmonary functions and capacities of firefighters based on sex, age-group and smoking status.

Pulmonary Functions and Capacities	Sex				Age-Group (Years)								Smoking Status			
	Males (n = 239)		Females (n = 39)		≤29 (n = 50)		30–39 (n = 93)		40–49 (n = 79)		≥50 (n = 56)		Non-Smokers (n = 176)		Smokers (n = 102)	
	n	%	n	%	n	%	n	%	n	n	%	n	%	n	%	n
Forced vital capacity																
Normal compliance	233	97.48	39	100	50	100	93	100	76	96.20	53	94.64	171	97.16	101	99.02
Borderline restriction	2	0.84	0	0.00	0	0.00	0	0.00	1	1.27	1	1.79	2	1.14	0	0.00
Slight or mild restriction	3	1.26	0	0.00	0	0.00	0	0.00	2	2.53	1	1.79	2	1.14	1	0.98
Moderate restriction	0	0.00	0	0.00	0	0.00	0	0.00	0	0.00	0	0.00	0	0.00	0	0.00
Severe restriction	1	0.42	0	0.00	0	0.00	0	0.00	0	0.00	1	1.79	1	0.57	0	0.00
Forced expiratory volume in one second																
Normal airflow	231	96.65	39	100	49	98.00	91	97.85	78	98.73	52	92.86	172	97.73	98	96.08
Borderline obstruction	1	0.42	0	0.00	0	0.00	0	0.00	0	0.00	1	1.79	0	0.00	1	0.98
Slight or mild obstruction	6	2.51	0	0.00	1	2.00	2	2.15	1	1.27	2	3.57	3	1.71	3	2.94
Moderate obstruction	1	0.42	0	0.00	0	0.00	0	0.00	0	0.00	1	1.79	1	0.57	0	0.00
Severe obstruction	0	0.00	0	0.00	0	0.00	0	0.00	0	0.00	0	0.00	0	0.00	0	0.00
Tiffeneau index																
Normal airflow	239	100	39	100	50	100	93	100	79	100	56	100	176	100	102	100
Borderline airflow limitation	0	0.00	0	0.00	0	0.00	0	0.00	0	0.00	0	0.00	0	0.00	0	0.00
Airflow limitation	0	0.00	0	0.00	0	0.00	0	0.00	0	0.00	0	0.00	0	0.00	0	0.00
Forced mid-maximal expiratory flow																
Normal airflow	158	66.11	18	46.15	29	58.00	55	59.14	52	65.82	40	71.43	110	62.50	66	64.71
Borderline obstruction	5	2.09	0	0.00	1	2.00	1	1.08	0	0.00	3	5.36	3	1.71	2	1.96
Slight or mild obstruction	48	20.08	17	43.59	14	28.00	23	24.73	22	27.85	6	10.71	41	23.30	24	23.53
Moderate obstruction	25	10.46	4	10.26	6	12.00	13	13.98	4	5.06	6	10.71	21	11.93	8	7.84
Severe obstruction	3	1.26	0	0.00	0	0.00	1	1.08	1	1.27	1	1.79	1	0.57	2	1.96
Peak expiratory flow rate																
Normal airflow	227	94.98	38	97.44	47	94.00	90	96.77	74	93.67	54	96.43	172	97.73	93	91.18
Borderline airflow obstruction	1	0.42	0	0.00	0	0.00	0	0.00	1	1.27	0	0.00	0	0.00	1	0.98
Airflow obstruction	11	4.60	1	2.56	3	6.00	3	3.23	4	5.06	2	3.57	4	2.27	8	7.84

Note: FVC: normal > 80% of predicted FVC; borderline restriction = 80% of predicted FVC; slight or mild restriction = 60–79% of predicted FVC; moderate restriction = 50–59% of predicted FVC; severe restriction < 50% of predicted FVC. FEV_1.0_: normal > 80% of predicted FEV_1.0_; borderline obstruction = 80% of predicted FEV_1.0_; slight or mild obstruction = 60–79% of predicted FEV_1.0_; moderate obstruction = 40–59% of predicted FEV_1.0_; severe obstruction < 40% of predicted FEV_1.0_. PEFR: normal ≥ 70% of predicted PEFR; reduced <70% of predicted PEFR.

**Table 3:** Differences in pulmonary functions and capacities of firefighters based on age-group.

	Sex				
Pulmonary Function Variables	Male (n = 239)		Female (n = 39)		
	($\bar{x}$ ± SD)		($\bar{x}$ ± SD)		*p*
FVC (L·min^−1^)	4.58 ± 0.89		3.36 ± 0.57		<0.001 **
FEV_1.0_ (L·min^−1^)	3.69 ± 0.70		2.75 ± 0.43		<0.001 **
FEV_1.0_/FVC (ratio)	0.82 ± 0.06		0.84 ± 0.05		<0.001 **
FEF_25–75_ (L·min^−1^)	3.98 ± 1.17		3.15 ± 0.74		<0.001 **
PEFR (L·min^−1^)	9.46 ± 1.90		6.98 ± 1.43		<0.001 **
	Age-group (years)				
Pulmonary Function Variables	≤29	30–39	40–49	≥50	
	(n = 50)	(n = 93)	(n = 79)	(n = 56)	
	($\bar{x}$ ± SD)	($\bar{x}$ ± SD)	($\bar{x}$ ± SD)	($\bar{x}$ ± SD)	*p*
FVC (L·min^−1^)	4.60 ± 0.74	4.73 ± 1.03	4.11 ± 0.94	4.11 ± 0.79	<0.001 **
FEV_1.0_ (L·min^−1^)	3.80 ± 0.64	3.80 ± 0.79	3.32 ± 0.68	3.31 ± 0.64	<0.001 **
FEV_1.0_/FVC (ratio)	0.84 ± 0.05	0.82 ± 0.06	0.83 ± 0.05	0.82 ± 0.07	0.211
FEF_25–75_ (L·min^−1^)	4.17 ± 1.24	3.95 ± 1.12	3.71 ± 1.05	3.66 ± 1.25	0.129
PEFR (L·min^−1^)	9.16 ± 2.05	9.42 ± 1.99	8.63 ± 1.97	9.24 ± 2.10	0.066

Note: ** indicates statistically significant differences *p* < 0.01. FVC = forced vital capacity; FEV_1.0_ = forced expiratory volume in 1 s; FEV_1.0_/FVC; FEF_25–75_ = forced mid-maximal expiratory flow; PEFR = peak expiratory flow rate.

**Table 4:** Differences in pulmonary functions and capacities of firefighters based on smoking status.

	Smoking Status		
Pulmonary Function Variables	Smokers	Non-Smokers	
	(n = 176)	(n = 102)	
	($\bar{x}$ ± SD)	($\bar{x}$ ± SD)	*p*
FVC (L·min^−1^)	4.35 ± 1.00	4.50 ± 0.87	0.101
FEV_1.0_ (L·min^−1^)	3.53 ± 0.78	3.63 ± 0.68	0.142
FEV_1.0_/FVC (ratio)	0.83 ± 0.06	0.82 ± 0.06	<0.001 **
FEF_25–75_ (L·min^−1^)	3.83 ± 1.13	3.92 ± 1.20	0.265
PEFR (L·min^−1^)	9.09 ± 2.01	9.15 ± 2.08	0.416

Note: ** indicates statistically significant differences *p* < 0.01. FVC = forced vital capacity; FEV_1.0_ = forced expiratory volume in 1 s; FEV_1.0_/FVC = Tiffeneau index; FEF_25–75_ = forced mid-maximal expiratory flow; PEFR = peak expiratory flow rate.

**Table 5:** Association between the pulmonary functions and capacities, and the smoking behaviour of firefighters.

Lung Function Variable	Smoking Status		No. of Cigarettes		Years Smoking	
	χ^2^	*p*	χ^2^	*p*	χ^2^	*p*
FVC	194.27	0.27	2002.80	0.003 **	1026.21	0.94
FEV_1.0_	180.63	0.35	1646.46	0.946	1051.28	0.431
FEV_1.0_/FVC	21.90	0.824	221.04	0.999	181.79	0.327
FEF_25–75_	193.33	0.710	1772.25	1.000	1157.46	0.930
PEFR	226.34	0.610	2466.21	0.025 *	1442.74	0.316

Note: * indicates statistically significant differences *p* < 0.05; ** indicates statistically significant differences *p* < 0.01; χ^2^ = Chi-squared test statistic. FVC = forced vital capacity; FEV_1.0_ = forced expiratory volume in 1 s; FEV_1.0_/FVC = Tiffeneau index; FEF_25–75_ = forced mid-maximal expiratory flow; PEFR = peak expiratory flow rate.

**Table 6:** Differences in pulmonary functions and capacities of firefighters based on BMI categories.

Pulmonary Function Variables	Body Mass Index Categories (kg·m^−2^)					*p*
	Healthy weight (18.5–24.9)	Overweight (25–29.9)	Grade 1 obesity (30–34.9)	Grade 2 obesity (35–39.9)	Grade 3 morbid obesity (≥40)	
	(n = 67)	(n = 115)	(n = 67)	(n = 20)	(n = 9)	
	($\bar{x}$ ± SD)	($\bar{x}$ ± SD)	($\bar{x}$ ± SD)	($\bar{x}$ ± SD)	($\bar{x}$ ± SD)	
FVC (L·min^−1^)	4.73 ± 0.91	4.56 ± 0.90	4.10 ± 0.91	3.89 ± 0.85	3.40 ± 0.89	<0.001 **
FEV_1.0_ (L·min^−1^)	3.82 ± 0.76	3.67 ± 0.70	3.33 ± 0.69	3.22 ± 0.62	2.68 ± 0.58	<0.001 **
FEV_1.0_/FVC (ratio)	0.82 ± 0.06	0.82 ± 0.06	0.83 ± 0.06	0.85 ± 0.05	0.82 ± 0.07	0.384
FEF_25–75_ (L·min^−1^)	3.95 ± 1.27	3.92 ± 1.18	3.78 ± 1.09	3.93 ± 0.89	2.98 ± 0.65	0.099
PEFR (L·min^−1^)	9.25 ± 2.13	9.24 ± 1.89	9.07 ± 2.26	8.70 ± 1.79	7.68 ± 1.19	0.110

Note: ** indicates statistically significant differences *p* < 0.01. FVC = forced vital capacity; FEV_1.0_ = forced expiratory volume in 1 s; FEV_1.0_/FVC = Tiffeneau index; FEF_25–75_ = forced mid-maximal expiratory flow; PEFR = peak expiratory flow rate.

**Table 7:** Differences in pulmonary functions and capacities of firefighters based on waist-to-hip ratio.

Pulmonary Function Variables	Waist-to-Hip Ratio		*p*
	Healthy	High risk	
	(n = 123)	(n = 155)	
	($\bar{x}$ ± SD)	($\bar{x}$ ± SD)	
FVC (L·min^−1^)	4.63 ± 0.99	4.22 ± 0.88	<0.001 **
FEV_1.0_ (L·min^−1^)	3.72 ± 0.78	3.43 ± 0.69	<0.001 **
FEV_1.0_/FVC (ratio)	0.82 ± 0.06	0.83 ± 0.06	<0.001 **
FEF_25–75_ (L·min^−1^)	3.89 ± 1.22	3.84 ± 1.11	<0.001 **
PEFR (L·min^−1^)	8.92 ± 2.06	9.27 ± 2.00	<0.001 **

Note: ** indicates statistically significant differences *p* < 0.01. FVC = forced vital capacity; FEV_1.0_ = forced expiratory volume in 1 s; FEV_1.0_/FVC = Tiffeneau index; FEF_25–75_ = forced mid-maximal expiratory flow; PEFR = peak expiratory flow rate; healthy WHR < 0.90 for males and <0.85 for females; high-risk WHR ≥ 0.90 for males and ≥0.85 for females.

**Table 8:** Relationship between the pulmonary functions and capacities and the physical characteristics of firefighters.

Variables	FVC	FEV_1.0_	FEV_1.0_/FVC	FEF_25–75_	PEFR	BMI	WC	WHR	BF%
FVC	--								
FEV_1.0_	0.95 **	--							
FEV_1.0_/FVC	−0.30 **	−0.01	--						
FEF_25–75_	0.38 **	0.62 **	0.65 **	--					
PEFR	0.58 **	0.62 **	0.02	0.46 **	--				
BMI	−0.36 **	−0.37 **	0.09	−0.13 *	−0.15 *	--			
WC	−0.05	−0.04	0.01	−0.01	−0.00	0.23 **	--		
WHR	−0.01	0.01	0.01	0.02	0.03	0.10	0.99 **	--	
BF%	−0.47 **	−0.48 **	0.10	−0.23 **	−0.30 **	0.79 **	0.19 **	0.08	--
MM	0.51 **	0.49 **	−0.13 *	0.26 **	0.47 **	0.23 **	0.06	0.02	−0.18 **

Note: * indicates a statistically significant correlation *p* < 0.05; ** indicates a statistically significant correlation *p* < 0.01. FVC = forced vital capacity; FEV_1.0_ = forced expiratory volume in 1 s; FEV_1.0_/FVC = Tiffeneau index; FEF_25–75_ = forced mid-maximal expiratory flow; PEFR = peak expiratory flow rate; BMI = body mass index; WC = waist circumference; WHR = waist-to-hip ratio; BF% = body fat percentage; MM = muscle mass.

**Table 9:** Relationship between the pulmonary functions and capacities and the behavioural risk factors of firefighters.

Variables	FVC	FVC %	FEV_1.0_	FEV_1.0_ %	FEV_1.0_/FVC	FEV_1.0_/FVC %	FEF_25–75_	FEF_25–75_ %	PEFR	PEFR %	YOS	PA
FVC	--											
FEV_1.0_	0.95 **	0.58 **	--									
FEV_1.0_/FVC	−0.29 **	−0.22 **	−0.02	0.18 **	--							
FEF_25–75_	0.40 **	0.19 **	0.63 **	0.53 **	0.66 **	0.70 **	--					
PEFR	0.61 **	0.29 **	0.64 **	0.35 **	0.01	0.13 *	0.46 **	0.31 **	--			
YOS	−0.21 **	−0.02	−0.25 **	0.07	−0.033	0.23 **	−0.11	0.19 **	−0.05	0.14 *	--	
PA	0.27 **	0.11	0.25 **	0.06	−0.13 *	−0.11	0.04	−0.05	0.17 **	0.05	−0.27 **	--
Ave. alc. units	−0.147 *	−0.10	−0.166 *	−0.10	0.06	0.09	0.03	0.05	−0.02	−0.00	0.15 *	−0.22 **

Note: * indicates statistically significant differences *p* < 0.05; ** indicates statistically significant differences *p* < 0.01. FVC = forced vital capacity; FEV_1.0_ = forced expiratory volume in 1 s; FEV_1.0_/FVC = Tiffeneau index; FEF_25–75_ = forced mid-maximal expiratory flow; PEFR = peak expiratory flow rate; YOS = years of service; PA = physical activity; Ave. alc. units = average number of alcohol units consumed.

**Table 10:** Linear association between pulmonary functions and capacities, physical characteristics, and behavioural risk factors of firefighters.

	Univariable Models ^a^			Multivariable Models ^b^		
	B	R^2^	*p*-Value	B	R^2^	*p*-Value
Model: FVC						
Age ^c^	−0.266	0.07	<0.001 **	−0.249	0.56	<0.001
Years of service	−0.189	0.04	0.002 **	−0.035	0.00	0.562
Body mass index	−0.364	0.13	<0.001 **	−0.147	0.24	0.014 *
Waist circumference	−0.045	0.00	0.452	-	-	-
Waist-to-hip ratio	−0.008	0.00	0.889	-	-	-
Bodyfat percentage	−0.473	0.22	<0.001 **	−0.300	0.27	<0.001 **
Muscle mass	0.506	0.26	<0.001 **	0.471	0.26	<0.001 **
Physical activity	0.204	0.04	<0.001 **	0.138	0.24	0.022 *
Number of cigarettes	0.071	0.01	0.235	-	-	-
Alcohol consumption	−0.147	0.02	0.048 *	0.076	0.22	0.306
Model: FEV_1_ (L·min^−1^)						
Age ^c^	−0.308	0.10	<0.001 **	−0.322	0.53	0.001 **
Years of service	−0.233	0.05	<0.001 **	0.033	0.53	0.733
Body mass index	−0.367	0.14	<0.001 **	−0.198	0.56	<0.001
Waist circumference	−0.044	0.00	0.469	-	-	-
Waist-to-hip ratio	0.001	0.00	0.990	-	-	-
Bodyfat percentage	−0.481	0.23	<0.001 **	−0.230	0.57	<0.001
Muscle mass	0.487	0.24	<0.001 **	−0.051	0.53	0.449
Physical activity	0.192	0.04	0.001 **	−0.283	0.53	0.244
Number of cigarettes	0.058	0.00	0.333	-	-	-
Alcohol consumption	−0.166	0.03	0.025 *	0.091	0.50	0.090
Model: FEV1.0/FVC (ratio)						
Age ^c^	−0.042	0.00	0.486	-	-	-
Years of service	−0.054	0.00	0.371	-	-	-
Body mass index	0.092	0.01	0.125	-	-	-
Waist circumference	0.007	0.00	0.914	-	-	-
Waist-to-hip ratio	0.012	0.00	0.843	-	-	-
Bodyfat percentage	0.095	0.01	0.113	-	-	-
Muscle mass	−0.132	0.02	0.028 *	0.119	0.06	0.211
Physical activity	−0.084	0.01	0.165	-	-	-
Number of cigarettes	−0.084	0.01	0.161	-	-	-
Alcohol consumption	−0.054	0.00	0.474	-	-	-
Model: FEF_25–75_ (L·min^−1^)						
Age ^c^	−0.180	0.03	0.003 **	−0.173	0.11	0.003 **
Years of service	−0.129	−0.02	0.032 *	0.095	0.11	0.479
Body mass index	−0.131	0.02	0.028 *	−0.483	0.11	0.629
Waist circumference	−0.009	0.00	0.879	-	-	-
Waist-to-hip ratio	0.022	0.00	0.713	-	-	-
Bodyfat percentage	−0.232	0.05	<0.001 **	−0.112	0.11	0.093
Muscle mass	0.260	0.07	<0.001 **	0.143	0.11	0.121
Physical activity	0.061	0.00	0.307	-	-	-
Number of cigarettes	0.033	0.00	0.584	-	-	-
Alcohol consumption	0.065	0.00	0.381	-	-	-
Model: PEFR (L·min^−1^)						
Age ^c^	−0.064	0.00	0.285	-	-	-
Years of service	−0.035	0.00	0.562	-	-	-
Body mass index	−0.147	0.02	0.014	−0.077	0.24	0.186
Waist circumference	−0.002	0.00	0.973	-	-	-
Waist-to-hip ratio	0.028	0.00	0.647	-	-	-
Bodyfat percentage	−0.300	0.09	<0.001	−0.191	0.27	0.002 **
Muscle mass	0.471	0.22	<0.001	0.244	0.26	0.004 **
Physical activity	0.138	0.02	0.022	0.065	0.24	0.230
Number of cigarettes	−0.003	0.00	0.955	-	-	-
Alcohol consumption	0.076	0.01	0.306	-	-	-

Note: * indicates statistically significant differences *p* < 0.05; ** indicates statistically significant differences *p* < 0.01. B—standardised beta coefficient; R^2^—coefficient of determination; FVC = forced vital capacity; FEV_1.0_ = forced expiratory volume in 1 s; FEV_1.0_/FVC = Tiffeneau index; FEF_25–75_ = forced mid-maximal expiratory flow; PEFR = peak expiratory flow rate; YOS = years of service; PA = physical activity; Ave. alc. units = average number of alcohol units consumed. ^a^—indicates univariable models; ^b^—indicates multivariable models adjusted for age and height; ^c^—indicates model that adjusted for years of service.

## Data Availability

The data presented in this study are available on request from the corresponding author. The data are not publicly available due to this being an ongoing study and the POPIA act, which restricts access to private information being made publicly available.
